# Trends of myopia development among primary and junior school students in the post-COVID-19 epidemic period

**DOI:** 10.3389/fpubh.2022.970751

**Published:** 2022-10-04

**Authors:** Wen Zhou, Qin Li, Hongyan Chen, Ya Liao, Wei Wang, Yifei Pei, Suyan Li, Wenxuan Zhang, Qian Wang, Xiaojuan Wang

**Affiliations:** ^1^Department of Ophthalmology, The First People's Hospital of Xuzhou, The Affiliated Xuzhou Municipal Hospital of Xuzhou Medical University, Xuzhou, China; ^2^Department of Community and Health Education, School of Public Health, Xuzhou Medical University, Xuzhou, China

**Keywords:** myopia development, post-COVID-19, spherical equivalent refraction, primary and junior students, epidemic

## Abstract

**Purpose:**

To investigate the trends of myopia among primary and junior school students in the post-COVID-19 epidemic period.

**Method:**

A prospective of cross-sectional study using spot photoscreenings in 123,538 children among primary and junior school students from 2019 to 2021 was conducted to evaluate the development of myopia in Xuzhou, China in the post-COVID-19 epidemic period. Equivalent refraction and the prevalence of myopia were recorded.

**Results:**

The spherical equivalent refraction of myopia decreased across all grades except grade 1 (0.23 ± 0.56 D in 2019, 0.24 ± 0.63 D in 2020) from 2019 to 2020. However, refraction exhibited a hyperopic shift in 2021 compared to 2020 for grades 1–5 (no significant decreased for grade 4). The prevalence of myopia in all grades increased in 2020 compared to 2019, and the most dramatic changes were observed from grades 2–5 and grades 7–8 (*P* < 0.05). The changes in myopia prevalence in grades 1–4 were mild, and the reduction in myopia for Grade 5 is significant from 2020 to 2021. Nevertheless, students in grades 6 and 9 exhibited the greatest growth in myopia prevalence (*P* < 0.01). All grades had higher myopia prevalence in 2021 compared with 2019, except grade 1 (*P* = 0.25). The prevalence of myopia in girls was higher compared with boys, and the urban myopia prevalence was higher than in rural areas over the 3 years except in 2019 (*P* = 0.18).

**Conclusions:**

The prevalence of myopia increased during the COVID-19 epidemic. However, the spherical equivalent refraction of lower grade children drifted to hyperopia and the trends of myopia development remained stable in the post-COVID-19 epidemic period. We should be more concerned about the prevalence of myopia in graduating for the primary or junior grades in the future.

## Introduction

Myopia is the most common type of refractive error, and its prevalence is increasing yearly. According to the current trends of progression, by 2050, ~50% (5 billion) of the global population will be afflicted by myopia (1). The rapid development of myopia requires timely treatment and prevention because it causes inconvenience to daily life, and the degree of myopia may be exacerbated if not treated. In addition, high myopia is often accompanied by eye diseases, such as retinal detachment, glaucoma, cataracts, posterior scleral staphyloma, and other complications that can lead to permanent visual impairment or even blindness in severe cases (2). Less outdoor activity and more near-work study time are common risk factors for myopia development (3).

An outbreak of viral pneumonia called coronavirus disease (COVID-19) has swept the country since mid-December 2019. Various containment measures were implemented nationwide, and a widespread “shutdown” of activities, including outdoor activities and school closures (4), occurred during the pandemic, to protect the health of young people eliminate virus transmission, and ensure the successful completion of education and teaching, people were forced to stay home in isolation. In addition, outdoor activities were banned for at least 3 months (from February to April), and students were required to study online using digital screen devices for 4 months (from February to May) (5). As online classes continue, students' exposure to electronic devices has increased significantly, seriously affecting their vision (6). Several studies have demonstrated increased myopia development in children during the COVID-19 epidemic (7, 8). Nevertheless, after strenuous efforts, the current epidemic prevention and control situation has been positively effective in China, allowing the school to open normally in September 2020. The country has now entered the “post-COVID-19 epidemic period” (9). Despite the measures taken to respond to the epidemic, the highly variable and rapid spread of the new coronavirus has created a great deal of uncertainty in the development of myopia among students in the post-COVID-19 epidemic period. The long-term persistence of the epidemic in the post-COVID-19 epidemic period may have a more pronounced effect on changes in myopia development and study behavior in our children and adolescents than the occasional outbreaks of the epidemic in the short term. In this study, we aimed to investigate trends in the development of myopia among Chinese students in the post-COVID-19 epidemic period.

## Methods

### Study population

According to the requirements of the “Comprehensive Prevention and Control of Myopia in Children and Adolescent Implementation Plan” (10), medical and health institutions in Xuzhou City started to conduct a comprehensive vision health screening work for primary and secondary schools under the guidance of the Provincial Health and Health Commission from 2019 and students in all grades were screened in the first 4 months of the new school year, from September to December. Cluster sampling was performed from each of the five districts in 2019, including Tongshan District, Quanshan District, Yunlong District, Jiawang District, and Gulou District. Then, the same schools selected in 2019 were also assessed in 2020 and 2021. In total, 123,728 students were selected for the study, of which 190 students were excluded due to missing eye data. Thus, a total of 123,538 students were included in the final analysis. Thirty-five primary schools and 10 junior schools were chosen for final analysis. Including 20 schools in the urban and 25 schools in the rural. Grades 1–6 were assessed in primary school, and grades 7–9 were assessed in junior high school.

This prospective of cross-sectional study was approved by the Ethics Board of The First People's Hospital of Xuzhou (No.xyyll[2019]022). The purpose and process were explained to all children and their parents before the start of the study. No compensation or rewards were offered for participation. Finally, all study procedures adhered to the Declaration of Helsinki.

### Measurements

The school was informed of the screening date 1 week in advance, and consent was obtained from the student and parents. Students who regularly wore contact lenses were instructed not to wear them at the time of the screening. Students undergoing corneal refractive therapy were also asked not to wear ortho-K lenses on the night before the screening and to wear their glasses on the screening date. Students were asked to remove their glasses for refractive testing.

The Spot photoscreener (Welch Allyn, VS100), a non-cycloplegic photorefrator, was used to measure the refractive status of students. Both eyes can be tested simultaneously through the visual stimulation of the screener and the sound played to attract the attention of the child. Testing was conducted by staff with extensive inspection experience. Due to the epidemic, staff and students wore masks during screening in 2020 and 2021, and the testing distance was approximately 1 m. A relative darkroom environment was chosen for screening, and different age patterns of Spot for students were selected according to their age. Then, the screener automatically generated the results. If severe refractive error, strabismus, or anisometropia was detected, the device would display a message advising the child to go to a specialized hospital for a complete eye examination.

### Definitions

The examination range of the Spot photoscreener was within ±7 D. If the outcome was beyond the ±7.50 D range, the value was recorded as ±8 D for further analysis. The spherical equivalent refraction (SER) was defined as the sum of the spherical refraction and one-half of the cylindrical refraction. If either eye of a student was myopic, the student was defined as having myopia. The severity of myopia was classified according to the following criteria: mild myopia (−3.00 D < SER≤ −0.50 D), moderate myopia (−6.00 D < SER≤ −3.00 D), high myopia (SER≤ −6.00 D) (7). Cases not meeting these criteria were classified as no myopia.

### Statistical analysis

Statistical analysis was performed using SPSS 23.0 (IBMSPSS, Armonk, NY, USA), Microsoft Excel 2010, and the figures were prepared using OriginPro 2021. One-way analysis of the variance of the means was used to compare categorical variables between groups. If *P* < 0.05, *post-hoc* LSD was used to compare the two groups. Both the chi-squared test and multiple comparisons were used to test for the prevalence of myopia in 2019, 2020, and 2021. Spearman's rank correlation coefficient was used to assess the correlation between the SER of the left and right eyes. Given that the SERs of both eyes were highly correlated (Spearman's rank correlation = 0.803, *P* < 0.001), we used the SER of the student's right eye as the basis for assessing myopia development and degree of myopia. *P* < 0.05 were considered statistically significant.

## Result

A total of 42,918 students in 2019, 41,964 students in 2020, and 38,656 students in 2021 were included in this study, with grades ranging from 1^st^-6th grade in primary school to 7^th^-9th grade in junior school. More detailed information about the number of observations for different grades and the distribution of sex and region over the 3 years are provided in Table 1. There were 2 students with SER > −8.00 D. No students had SER > +8.00 D in at least 1 eye.

**Table 1:** The distribution of populations with different demographic parameters.

		**2019**	**2020**	**2021**
**Grade**				
Primary school	1	5,149 (11.9)	4,527 (10.8)	4,041 (10.5)
	2	5,234 (12.2)	4,988 (11.9)	4,049 (10.5)
	3	5,084 (11.8)	5,164 (12.3)	4,500 (11.6)
	4	4,981 (11.6)	5,365 (12.8)	4,554 (11.8)
	5	5,293 (12.3)	5,418 (12.9)	4,143 (10.7)
	6	5149 (12.0)	5,403 (12.9)	4,673 (12.1)
Junior school	7	4,821 (11.2)	4,343 (10.3)	4680 (12.1)
	8	3,883 (9.0)	3,689 (8.8)	4,256 (11.0)
	9	3,369 (7.8)	3,067 (7.3)	3,760 (9.7)
**Sex**				
Male		23,797 (55.4)	23,114 (55.1)	21,267 (55.0)
Female		19,121 (44.6)	18,850 (44.9)	17,389(45.0)
**Region**				
Urban		23,916 (55.7)	25,159 (60.0)	20,893 (54.0)
Rural		19,002 (44.3)	16,805 (40.0)	17,763 (46.0)
**Total**		42,918	41,964	38,656

**Figure 1:**
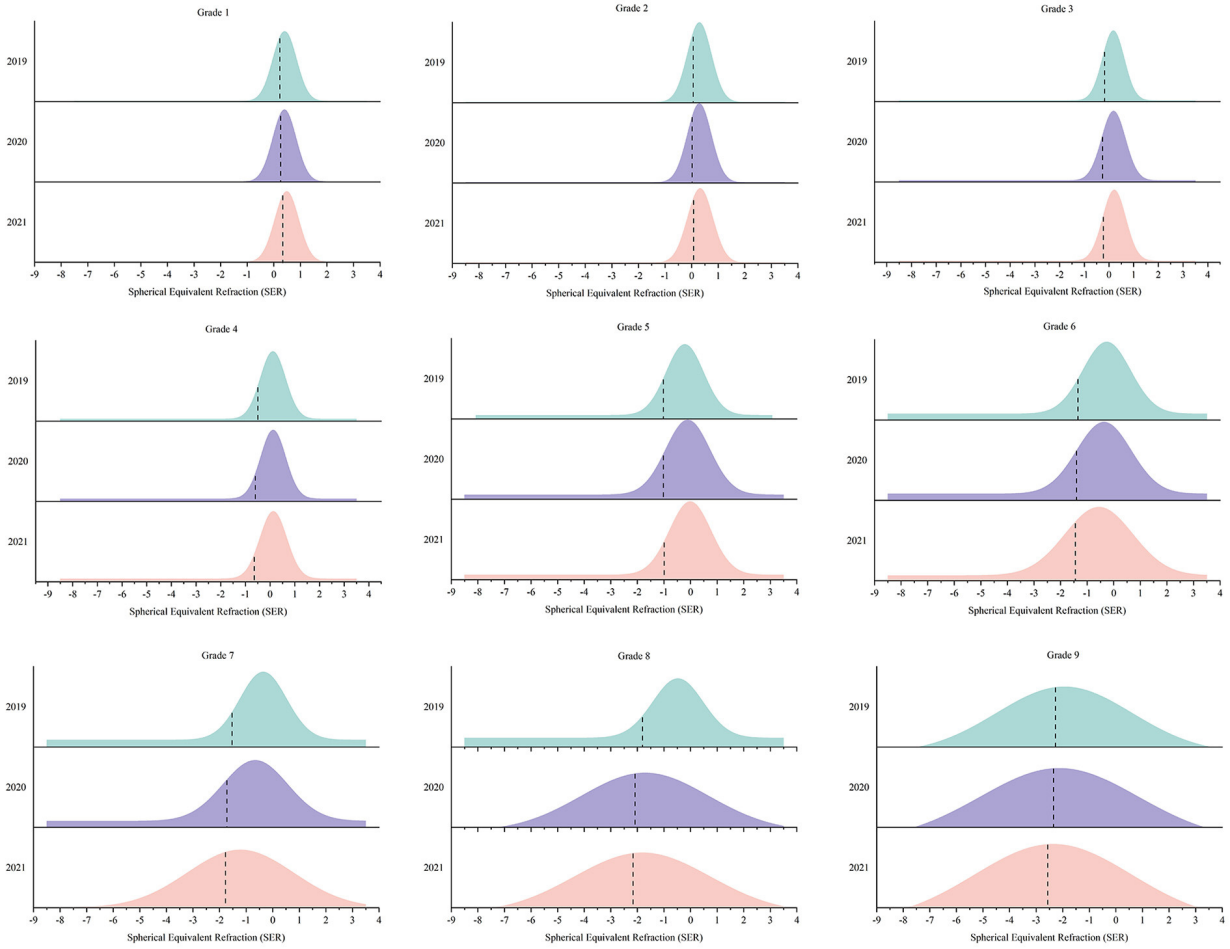
The distribution of Spherical Equivalent Refraction (SER) from grades 1 to grade 9 over the 3 years.

The mean SER from 2019 to 2021 is shown in Table 2. Except for grade 1 (0.24 ± 0.63 D in 2020, 0.23 ± 0.56 D in 2019), the SER for other grades decreased to varying degrees from 2019 to 2020. The greatest decrease in SER was observed in grades 3–5 and 7–9 (*P* < 0.01). However, almost all the mean SER for grades 1–5 exhibited hyperopic shifts in 2021 compared to 2020 (insignificant reduction of SER in grade 4). Grades 6 and 9 had the highest decrease in SER in 2021 compared to 2020 (*P* < 0.05). The mean SER decreased in 2021 compared to 2019 for all grades except grade 1. To demonstrate the distribution of SER from grades 1 to 9 over the 3 years, Gaussian fitting curves based on the frequency histogram are displayed in Figure 1.

**Table 2:** SER values from grades 1 to grade 9 over the 3 years.

		**SER, mean**			
**Grade**		**2019**	**2020**	**2021**	***P*** **-value ^a^**	***P*** **-value ^b^**	***P*** **-value ^c^**
**Primary school**	1	0.23 ± 0.56	0.24 ± 0.63	0.32 ± 0.71	0.52	<0.01^*^	<0.01^*^
	2	0.05 ± 0.758	0.03 ± 0.85	0.05 ± 0.93	0.30	0.92	0.39
	3	−0.21 ± 0.98	−0.28 ± 1.11	−0.25 ± 1.15	<0.01^*^	0.07	0.17
	4	−0.52 ± 1.25	−0.62 ± 1.33	−0.67 ± 1.40	<0.01^*^	<0.01^*^	0.06
	5	−0.93 ± 1.50	−1.04 ± 1.59	−1.01 ± 1.60	<0.01^*^	0.01^*^	0.39
	6	−1.35 ± 1.68	−1.41 ± 1.76	−1.49 ± 1.81	0.09	<0.01^*^	0.01^*^
**Junior school**	7	−1.54 ± 1.75	−1.72 ± 1.85	−1.77 ± 1.94	<0.01^*^	<0.01^*^	0.20
	8	−1.84 ± 1.88	−2.13 ± 1.94	−2.17 ± 2.03	<0.01^*^	<0.01^*^	0.31
	9	−2.28 ± 2.02	−2.35 ± 2.04	−2.57 ± 2.05	0.18	<0.01^*^	<0.01^*^

* Significance was set at 0.05.

One-way analysis of variance of the means was used to compare categorical variables between groups.

a, b and c represent P-values for using *Post-Hoc* Multilpe Comparisons between 2019 and 2020, 2019 and 2021, 2020 and 2021, respectively.

The prevalence of myopia from 2019 to 2021 in grades 1–9 is presented in Table 3. From 2019 to 2020, the prevalence of myopia for all grades increased, and the most dramatic change was observed from grades 2–5 and grades 7–8 (*P* < 0.05). Grade 5 had the largest increase in myopia prevalence (5.6%), followed by grades 8 (5.1%), 3 (4.6%), and 7 (4.3%), all of which had an >3.0% increase in prevalence. From 2020 to 2021, the changes in myopia in grades 1–4 were mild and not significantly different. Students in grade 5 had the greatest reduction in myopia (*P* = 0.01). Nevertheless, students in grades 6 and 9 had the greatest growth in myopia prevalence (*P* < 0.01). Prevalence of myopia were higher for all grades in 2021 except grade 1 (*P* = 0.25).

**Table 3:** Prevalence of myopia (SER ≤ −0.5D) from grades 1 to grade 9 over the 3 years.

		**Prevalence per year, %**			
**Grades**		**2019**	**2020**	**2021**	***P*** **-value^a^**	***P*** **-value^b^**	***P*** **-value^c^**
**Primary school**	1	6.8	7.1	6.2	0.47	0.25	0.07
	2	16.0	18.3	19.1	0.01^*^	<0.01^*^	0.33
	3	29.4	34.0	33.0	<0.01^*^	<0.01^*^	0.29
	4	45.0	47.7	49.5	0.01^*^	<0.01^*^	0.07
	5	56.8	62.4	59.9	<0.01^*^	0.01^*^	0.01^*^
	6	68.1	69.7	72.5	0.08	<0.01^*^	0.01^*^
**Junior school**	7	72.1	76.4	77.1	<0.01^*^	<0.01^*^	0.44
	8	77.0	82.1	82.6	<0.01^*^	<0.01^*^	0.54
	9	82.7	83.3	88.0	0.52	<0.01^*^	<0.01^*^

* Significance was set at 0.05.

The Chi-square test was used to compare categorical variables between groups.

a, b and c represent P-values for Multiple Comparisons between 2019 and 2020, 2019 and 2021, 2020 and 2021, respectively.

**Figure 2:**
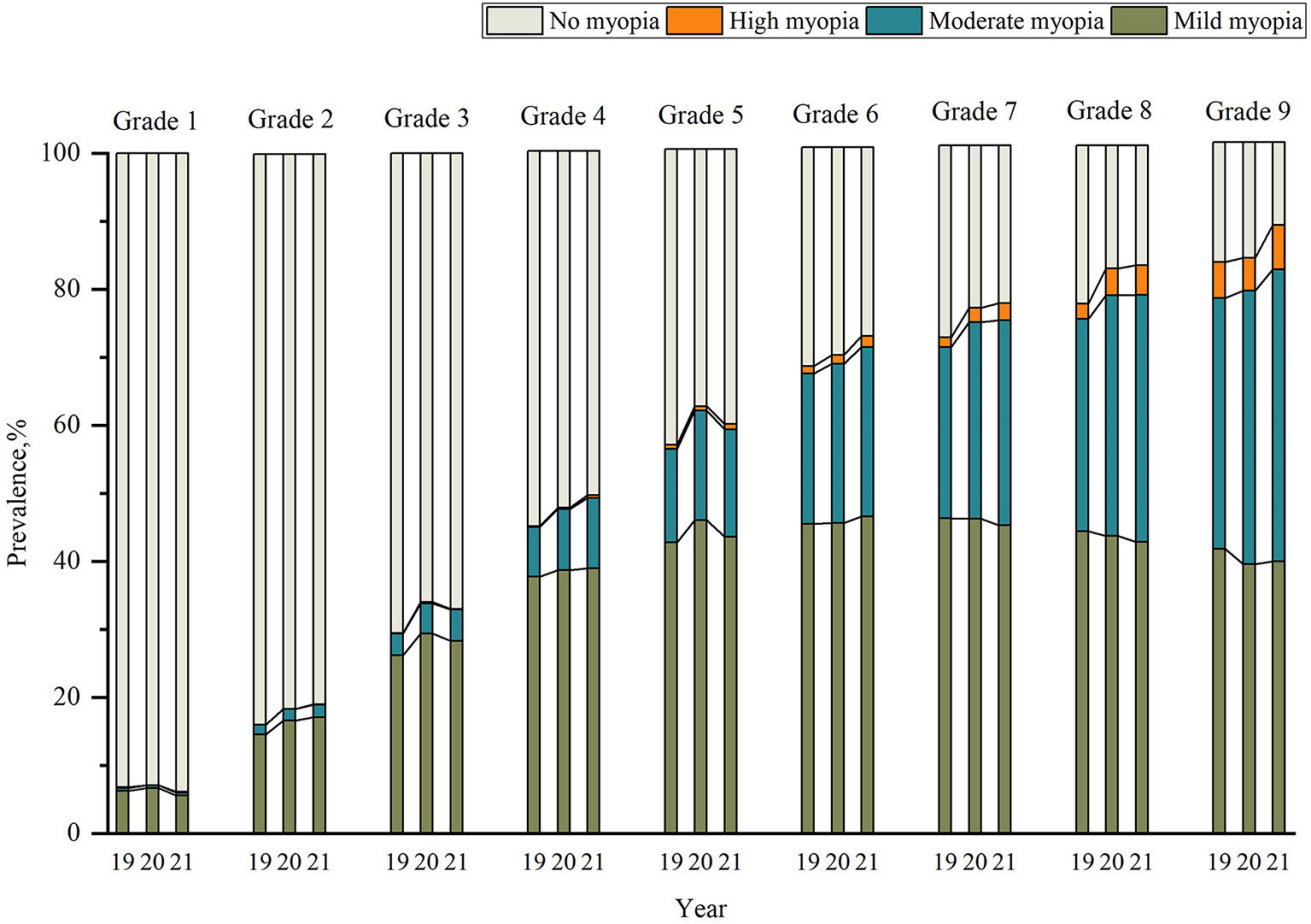
Changes in the degree of myopia in grades 1–9 over the 3 years.

Further analysis showed that the mean SER in urban areas was greater than in rural areas over 3 years except in 2019 (*P* = 0.18), whereas the females were more myopic than males over the 3 years (Table 4). Chi-square tests for trend revealed that the amount of mild myopia increased as the grade level increased from grades 1–6, and gradually decreased with increasing grades from grades 7–9 over 3 years (*P* < 0.001) (Table 5). The prevalence of moderate myopia increased gradually from grades 1–9 over 3 years.The proportion of severe myopia remained stable from Grade 1 to grade 4 but gradually increased from grade 5 (Table 5, Figure 2).

**Table 4:** SER values stratified by gender and region over 3 years.

	**SER, mean**				
	**2019**	**2020**	**2021**	**F**	***P*** **^e^**
Male	−0.79 ± 1.60	−0.87 ± 1.69	−0.98 ± 1.80	74.82	<0.001
Female	−0.92 ± 1.65	−1.02 ± 1.73	−1.16 ± 1.88	84.00	<0.001
t	8.27	8.93	9.21		
*P*^d^	<0.001	<0.001	<0.001		
Urban	−0.84 ± 1.63	−0.96 ± 1.72	−1.11 ± 1.87	139.70	<0.001
Rural	−0.86 ± 1.62	−0.89 ± 1.69	−1.00 ± 1.80	34.76	<0.001
t	1.35	−4.02	−5.84		
*P*^d^	0.18	<0.001	<0.001		

d Comparison underwent Independent Samples T Test.

e Comparison underwent One-way ANOVA, P-values of LSD were all < 0.05.

**Table 5:** The percentage of different degrees of myopia from grades 1 to grade 9 over the 3 years.

**Grades**	**2019**				**2020**				**2021**			
	**No myopia**	**Mild myopia**	**Moderate myopia**	**Severe myopia**	**No myopia**	**Mild myopia**	**Moderate myopia**	**Severe myopia**	**No myopia**	**Mild myopia**	**Moderate myopia**	**Severe myopia**
Grade 1	93.2%	6.3%	0.4%	0.1%	92.9%	6.7%	0.4%	0.0%	93.8%	5.6%	0.4%	0.1%
Grade 2	84.0%	14.6%	1.4%	0.0%	81.7%	16.6%	1.7%	0.0%	80.9%	17.1%	1.8%	0.1%
Grade 3	70.6%	26.2%	3.2%	0.1%	66.0%	29.4%	4.4%	0.2%	67.0%	28.3%	4.6%	0.1%
Grade 4	55.0%	37.6%	7.3%	0.1%	52.3%	38.6%	8.9%	0.2%	50.5%	38.9%	10.3%	0.4%
Grade 5	43.2%	42.5%	13.7%	0.6%	37.6%	45.8%	16.1%	0.6%	40.1%	43.3%	15.7%	0.8%
Grade 6	31.9%	45.1%	21.9%	1.1%	30.3%	45.3%	23.2%	1.3%	27.5%	46.2%	24.7%	1.6%
Grade 7	27.9%	45.8%	24.9%	1.4%	23.6%	45.7%	28.6%	2.1%	22.9%	44.8%	29.8%	2.5%
Grade 8	23.0%	43.9%	30.9%	2.2%	17.9%	43.2%	35.0%	3.9%	17.4%	42.3%	35.9%	4.3%
Grade 9	17.3%	41.1%	36.3%	5.2%	16.7%	39.0%	39.6%	4.8%	12.0%	39.4%	42.3%	6.4%
χ^2^	13,326.301				13,163.838				13,343.917			
P^*^	<0.001				<0.001				<0.001			

* Significance was set at 0.05.

## Discussion

In this survey, we reported changes in the development of myopia among primary and junior school students before and after the COVID-19 epidemic in Xuzhou, China. The Spot photoscreener used in this investigation showed good diagnostic accuracy and consistency in children's refractive errors screening (11, 12). The SE values obtained by the screener showed good agreement before and after ciliary muscle paralysis in students aged 7–18 years, and the difference in SE values between the autorefractor and Spot screener was ~ −0.3 D for both the mild and moderate myopia groups (13). Although Spot's accuracy was not as good as cycloplegic refraction, it is useful and effective for screening and monitoring myopia in large populations.

During the COVID-19 pandemic, primary and junior school students in Xuzhou were restricted to home and online education to ensure the successful completion of education and teaching. Our findings showed that the mean SER declined fastest in grades 3–5 and grades 7–8 in 2020 compared with 2019. In other grades, the change was not significant. This finding was slightly different from the results that also used the Spot photoscreener, which indicated the fastest change in SER was noted among children aged 6–8 years from 2019 to 2020 (7). Previous studies have confirmed accelerated myopic progression and more negative SER change during the COVID-19 pandemic (6, 7, 14–17), and we hypothesized that refraction may change in the post-COVID-19 epidemic period. Surprisingly, in this study, except for grades 6 and 9, the refraction of students in other grades remained almost the same in 2021 compared to 2020 and even drifted toward hyperopia in some cases. This finding may be explained by the high pressure experienced by the graduating classes for the primary and junior grades and the stress of offline teaching compared with online teaching.

The mean SER in grades 1 and 2 was higher than that in other reports (18, 19). Xuzhou city is located in the north of Jiangsu Province, which is an economically underdeveloped area with a relatively high proportion of rural areas. The pressure of schoolwork is not as high in this area compared with other developed areas, resulting in a lower myopia rate compared with other developed areas. In addition, the use of a Spot photoscreener in non-ciliary muscle paralysis optometry and examination at a farther distance leads to reduced accommodation stimulation. Thus, the refraction is more hyperopic compared with an autorefractor. In contrast, the stimulation of near-perceived accommodation of autorefractor is evident in non-myopic children. Therefore, the result trends toward myopia, but the effect of accommodation is not evident for myopes. Thus, this setup has minimal effect on the results of examinations in upper grades.

The development of myopia in students is closely related to lifestyle changes, and the prevalence of myopia is increasing yearly, especially in China (20). Asian countries are under more academic pressure and have a higher incidence of student myopia than Western countries. A 5-year longitudinal follow-up study of 6–15 years old students in Chongqing showed an annual prevalence of myopia of 10.6% (21). However, the results of a German survey of children aged 0–17 years over the last decade years showed minimal change in the prevalence of myopia (22). In our study, the prevalence of myopia among primary and junior school students increased between 2019 and 2020, and the prevalence of myopia increased with grade level. The increase in myopia prevalence was greater in grades 3–5 and 7–8. Li et al. found that the prevalence of myopia was lower before grade 3 (23), which is consistent with our findings. In contrast, compared to 2020, myopia rates in grades 1–5 were minimally changed in 2021, and even myopia reversal was observed. Myopia drifted to hyperopia after lockdown, as reported by Chang et al. (24). We supposed that this may be explained by accommodation spasms. The refractive system of the human eye is mainly composed of corneal curvature, eye axis length, and eye accommodation. Corneal curvature develops steadily after 2 years of age (25), and Ma et al. (16). reported no significant difference in the change of eye axis during the COVID-19 epidemic. Due to COVID-19, students were restricted to online learning at home, increasing digital screen time, reducing outdoor activities, and leading to accommodation spasms and consequent progression of myopia. However, when offline learning resumes in 2021, the accommodation spasms reverse and cause the refractive state to drift toward hyperopia. Therefore, we believe that partial reversal of the accommodation spasm can change the refractive state. In this study, children in the lower grades were experiencing a highly plastic period of myopia development, and myopia prevention and control may be more effective during this period. This notion should be further evaluated in additional studies. In addition, to guide the prevention and control of myopia among children and adolescents during the COVID-19 epidemic, the National Health Commission of the People's Republic of China has developed myopia prevention guidelines (26). These guidelines included how to provide myopia prevention after online learning and resumption of classes and emphasized reducing time spent on electronics and increasing outdoor activities. This policy contributed to a lower increase in students' myopia rates in 2021 than in 2020.

In our study, the prevalence of myopia in grade 1 was not reduced from 2019 to 2021, and was not affected by the COVID-19 epidemic. Because our annual screenings were conducted in the first 2 months of the school year, students in grade 1 were not truly in the learning stage. Thus, the prevalence of myopia was lower than in other grades.

The prevalence of myopia from grades 7–8 also increased in 2021 but was not statistically significant. However, the change in myopia rates for grades 6 and 9 was higher in 2021 than in 2020. The increased intensity of education in the graduating grades led to more time spent performing near-work activities, less time spent outdoors, and less time sleeping compared to the non-graduating grade. Educational intensity (27), outdoor activity time (28), and sleep duration (29) are associated with myopia progression. Offline courses were resumed in April 2020 (30). To compensate for the knowledge not covered by online education, graduating grades faced more pressure to pursue further education with stronger supervision from schools and teachers, leading to a surge in their learning pressure and a sharp increase in myopia. We recommend that schools and parents pay more attention to graduating grades during the post-COVID-19 epidemic period.

As shown in other studies (7, 14, 15), in our three-year study, females had higher myopia rates than males. This finding may be explained by the notion that girls are quieter than boys and spend more time studying and less time participating in outdoor activities than boys. An epidemiological study reports female is a risk factor for myopia (31). An article monitoring refractive development in children found that girls have steeper corneas, shallower anterior chambers (32), and shorter eye axes than boys (33). The present study noted that gender differences occurred primarily after grade 3 (Figure 2). Some studies suggest that this founding is due to estrogenic changes that alter female vision during adolescence (34). In addition, this study also confirmed that urban students have higher myopia rates than rural students over the 3 years except in 2019. During the epidemic period, the urban students were required to be segregated at home, while the rural students may be in the yard or in a more open area for activites. This phenomenon persisted in the post-COVID-19 epidemic. This result is comparable to the results of previous studies (35, 36). This finding suggests that environmental factors may play a major role in the development of myopia in Chinese children (37). However, more urban students were analyzed in higher numbers than the rural students over 3 years which could have also contributed to the gross increase in the number in raw data rather than a “true” increase in incidence, and further investigation is needed in future studies.

This study had some limitations. First, the myopia assessment index selected in this study was collected using the Spot photoscreener. Although this technology is useful for screening, it is currently not a substitute for cycloplegic refraction. Second, studies have shown that myopia is caused by a variety of factors, including light and time spent performing outdoor activities and near-work activities (38). Third, we did not include preschoolers and could not understand changes in the development of myopia in younger children in the post-COVID-19 epidemic period. Fourth, our study only analyzed the development of myopia according to gender and region. Subsequent studies should be designed more specifically to assess the factors influencing myopia development.

## Conclusion

Our research confirmed the prevalence of myopia increased during the COVID-19 epidemic. However, the spherical equivalent refraction of lower grades children drifted to hyperopia and the trends of myopia development remained stable in the post-COVID-19 epidemic period. We should be more concerned about the prevalence of myopia in graduating for the primary or junior grades in the future.

## Data availability statement

The original contributions presented in the study are included in the article/supplementary material, further inquiries can be directed to the corresponding author.

## Ethics statement

Study approval was obtained from the Ethics Board of The First People's Hospital of Xuzhou (No.xyyll[2019]022). Written informed consent to participate in this study was provided by the participants' legal guardian/next of kin.

## Author contributions

WZho and XW: concept and design. WZho, QL, HC, and YL: acquisition, analysis, and interpretation of data. WZho, QL, and SL: drafting of the manuscript. YL and XW: critical revision of the manuscript for important intellectual content. WZho, HC, WW, and YP: statistical analysis. XW: obtained funding and supervision. WZha and QW: administrative, technical, and material support. All authors contributed to the article and approved the submitted version.

## Funding

This study was supported by Xuzhou Medical Leading Talent Training Project (Grant No. XWRCHT20210022), Postgraduate Research and Practice Innovation Program of Jiangsu Province (Grant No. KYCX22_2978).

## Conflict of interest

The authors declare that the research was conducted in the absence of any commercial or financial relationships that could be construed as a potential conflict of interest.

## Publisher's note

All claims expressed in this article are solely those of the authors and do not necessarily represent those of their affiliated organizations, or those of the publisher, the editors and the reviewers. Any product that may be evaluated in this article, or claim that may be made by its manufacturer, is not guaranteed or endorsed by the publisher.
